# Photoreceptor Sensory Cilium: Traversing the Ciliary Gate

**DOI:** 10.3390/cells4040674

**Published:** 2015-10-15

**Authors:** Hemant Khanna

**Affiliations:** Department of Ophthalmology, UMASS Medical School, Worcester, MA 01605, USA; E-Mail: hemant.khanna@umassmed.edu; Tel.: +1-508-856-8991; Fax: +1-508-856-1552.

**Keywords:** cilia, flagella, ciliopathies, retina, retinopathies, transition zone, protein trafficking

## Abstract

Cilia are antenna-like extensions of the plasma membrane found in nearly all cell types. In the retina of the eye, photoreceptors develop unique sensory cilia. Not much was known about the mechanisms underlying the formation and function of photoreceptor cilia, largely because of technical limitations and the specific structural and functional modifications that cannot be modeled *in vitro*. With recent advances in microscopy techniques and molecular and biochemical approaches, we are now beginning to understand the molecular basis of photoreceptor ciliary architecture, ciliary function and its involvement in human diseases. Here, I will discuss the studies that have revealed new knowledge of how photoreceptor cilia regulate their identity and function while coping with high metabolic and trafficking demands associated with processing light signal.

## 1. Introduction

Light detection and processing dictate most of our memories of the world around us. The eye is the most exposed part of the brain and amenable to several manipulations that allow us to understand the deeper mysteries of the brain. Nonetheless, we know relatively little about the mechanisms by which we detect and process light signal. In fact, much greater part of our central nervous system (almost half of the cerebral cortex) is devoted to visual processing than any other system [1]. The detection of light is initiated as the light signal enters the anterior part of the eye and reaches the retina in the back of the eye. Here, the light is converted into a chemical signal that is transmitted through secondary order neurons to retinal ganglion cells. Axons of ganglion cells form the optic nerve, which convey the signal to the visual cortex in the brain. As first responders to light, photoreceptors have been the subject of several studies aimed at understanding the mode of light detection and processing. In recent years, a lot of progress has been made in understanding how photoreceptors develop unique structural modifications to process and enhance the light signal. In this review, I will focus on one such modification, which is the development and maintenance of the sensory compartment of photoreceptors, called the outer segment (OS).

## 2. Retina

**Figure 1:**
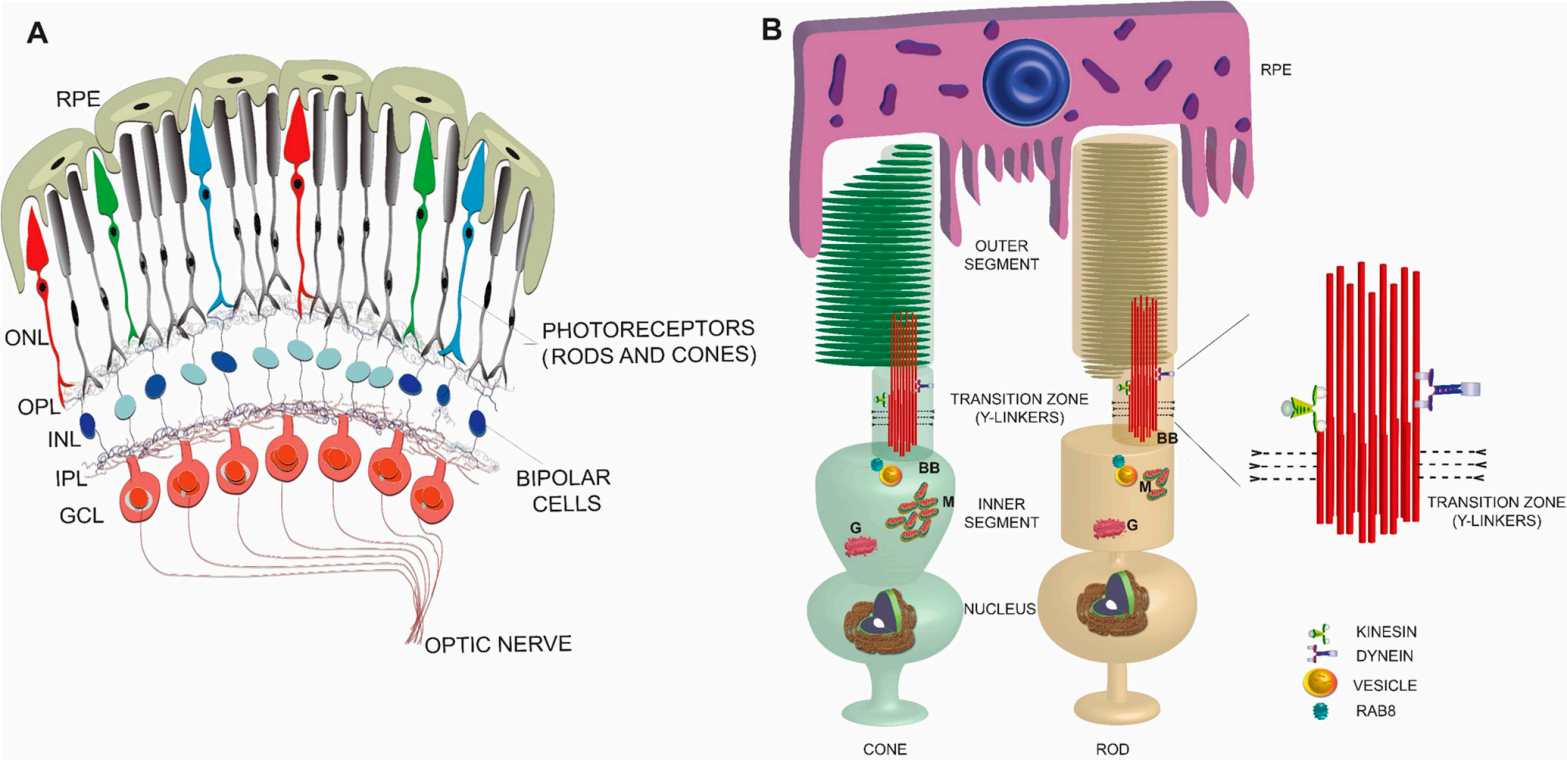
Schematic representation of a simplified view of mammalian retina (**A**) and rod and cone photoreceptors (**B**). An enlarged view of the TZ is shown on the right. RPE: retinal pigmented epithelium; ONL: outer nuclear layer; OPL: outer plexiform layer; INL: inner nuclear layer; IPL: inner plexiform layer; GCL: ganglion cell layer; BB: basal body; M: mitochondria; G: Golgi.

The retina is ~0.5 mm thick tissue situated in the back of the eye and is involved in the first steps of light sensation. It is a highly organized tissue consisting of six major types of neurons and one glial cell type separated by two synaptic layers, called the outer and the inner plexiform layers (Figure 1A). Among the neurons, photoreceptors are the most abundant cell types and form the outermost layer of the retina [1,2]. The tips of the photoreceptors are physically closest to the retinal pigment epithelium (RPE), which forms the outermost blood-retina barrier and is also involved in visual cycle and periodic maintenance of the photoreceptor sensory compartment. The choroidal blood vessels overlaying the RPE supply nutrients to photoreceptors.

## 3. Photoreceptors

Photoreceptors are highly polarized and metabolically active neurons with a distinct compartment, called the OS to house the phototransduction machinery. The OS is a modified sensory cilium, which contains membranous discs arranged in a coin-stack like fashion (Figure 1B). This elegantly complex structure is devoid of any protein translation machinery; hence, the components that populate the OS are synthesized in the inner segment (IS), which contains all the necessary organelles, including the endoplasmic reticulum (ER), Golgi, and mitochondria, and transported to the distal OS. Distal to the inner segment is the cell body containing the nucleus and synaptic termini that extend into the outer plexiform layer where they synapse with the second order neurons called bipolar cells [3].

## 4. Photoreceptor Sensory (or Primary) Cilium

Primary cilia are microtubule-based extensions of the apical plasma membrane and help in concentrating specific signaling receptors involved in modulating developmental signaling events, such as sonic hedgehog signaling, Wnt signaling, and platelet derived growth factor signaling. Cilia are also involved in modulating the signaling cascades involved in sensory perception, such as chemosensation, olfaction, mechanosensation, and photoreception (subject of this article) [4,5,6,7,8,9]. Consistent with a widespread involvement of ciliary function, ciliary proteins are associated with several human disorders, such as cystic kidney disease, retinal degeneration and pleiotropic genetic diseases Bardet-Biedl Syndrome (BBS), Joubert Syndrome, Senior-Loken Syndrome, Usher Syndrome, and Meckel-Gruber Syndrome [10,11,12,13,14].

Cilia in photoreceptors develop unique characteristics that help them adapt to the high demands of detecting light signals throughout the life of the organism. Elegant ultrastructural studies by Sjöstrand, De Robertis, and Tokuyasu and Yamada identified membranous discs in the OS of rod and cone photoreceptors [15,16,17,18,19]. In fact, the cilium was initially identified between the inner and the outer segments as the only connection between these components. Thus, this structure was named the connecting cilium [15,16,17,18,19]. The ciliary compartment of photoreceptors is loaded with proteins involved in the phototransduction cascade, such as the visual pigment opsin (a G-protein coupled receptor), transducin, arrestin, cGMP-phosphodiesterases, and cyclic nucleotide gated (CNG) channel. In addition, the photoreceptor OS are preferentially enriched in insulin growth factor receptor, Phosphoinositide 3-Kinase (PI3K) and AKT (AK-Transforming; serine threonine protein kinase B) signaling components [20] and in omega-3 docosahexaenoic acid (DHA), which is thought to provide fluidity to rhodopsin in the disc membranes. In turn, such functions regulate the fast phototransduction cascade that is ensued upon light detection [21,22,23].

Photoreceptor cilia also exhibit unique functional properties. This is because phototransduction is carried out in distinct compartments of photoreceptor cilia and utilizes both the disc membranes, as well as ciliary plasma membrane. In addition, the phototransduction cascade is regulated by the overlaying RPE. Hence, there is continuous flow of information and molecules within and out of the cilium.

## 5. Photoreceptor Ciliogenesis

**Figure 2:**
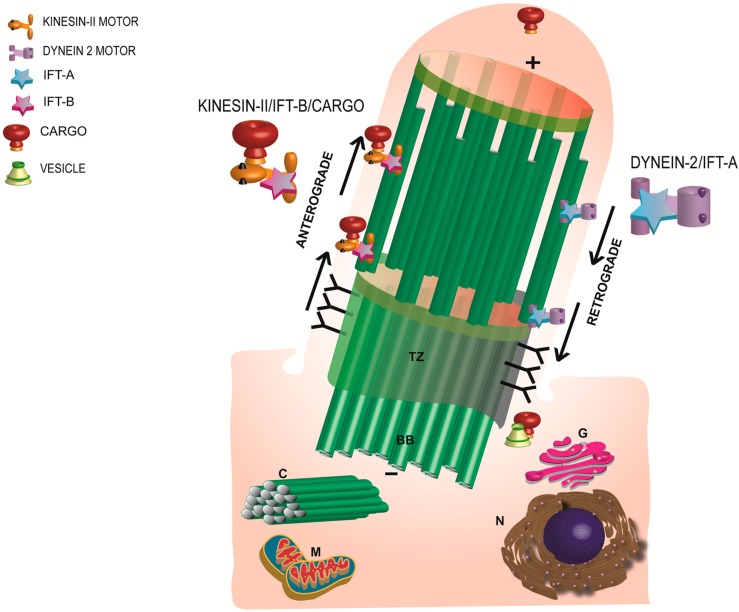
Schematic representation of the morphology and major components of a primary cilium. Anterograde transport is mediated by Kinesin-II motor and the IFT-B complex whereas the retrograde trafficking is regulated by the IFT-A and the dynein motor subunit. TZ: transition zone; BB: basal body; C: daughter centriole; G: Golgi; N: nucleus; M: mitochondria.

Ciliogenesis (or cilia formation) initiates when the mother centriole (also called basal body) consisting of an array of nine triplet microtubules docks at the apical plasma membrane and nucleates the extension of a doublet microtubule cytoskeleton called axoneme [24,25,26,27,28]. This elaborate process is governed by Intraflagellar Transport (IFT). First identified in *Chlamydomonas* flagella, IFT is defined as bidirectional transport of cargo from the base of the cilia to the tip (anterograde; plus end directed) and back to the base (retrograde; minus-end directed). The plus and minus ends refer to the growing and the nucleating ends of the microtubules from the basal bodies, respectively [29]. The IFT is powered by molecular motors kinesin-II (anterograde) and cytoplasmic dynein 2 (retrograde) and is divided into two major complexes: IFT-A and IFTB. Whereas IFT-B complex facilitates anterograde trafficking of the cargo, IFT-A is predominantly involved in retrograde trafficking [29,30] (Figure 2).

First evidence for the involvement of IFT in photoreceptor ciliogenesis and ciliary trafficking came from the analysis of a hypomorphic mouse mutant of *Ift88^orpk^*, which carries a mutation in IFT88, an IFT subunit. It was found that these mice have defective OS development with no evidence of ciliary extension [31]. Later studies also identified a zebrafish mutant of *ift88*, called *ovl*, which does not exhibit rod cilium generation. More recent studies have assessed the role of other IFT subunits in photoreceptor ciliogenesis [32]. Some IFT proteins, including IFT57, IFT52, IFT140, and IFT20 have been localized to photoreceptor cilia [33,34,35,36,37]. Moreover, it was found that ablation of IFT20 or IFT140 results in opsin mislocalization and photoreceptor degeneration. Interestingly, IFT20 is the only IFT subunit, which is also found at the Golgi [38]. It was shown that whereas deletion of *Ift20* results in the accumulation of opsin around the Golgi, *Ift140* deletion resulted in the predominant trafficking of rhodopsin to the plasma membrane of the inner segment rather than OS. As IFT140 belongs to IFT-A whereas IFT20 belongs to IFT-B, these studies indicate distinct roles of the two IFT complexes in maintaining cilia function in photoreceptors [38,39]. Additional evidence of the involvement of IFT in photoreceptor ciliogenesis came from the studies examining the involvement of IFT motors Kinesin-II and dynein subunits. Conditional deletion of a kinesin-II subunit KIF3A disrupts OS formation and opsin trafficking. It was recently found that embryonic deletion of *Kif3a^−/−^* in mice abrogates extension of the TZ from the basal bodies [40,41].

## 6. Photoreceptor Ciliary Trafficking

To maintain the identity of the OS, stringently controlled mechanisms are employed to regulate directional trafficking of proteins. Being indispensable for OS formation and function, rhodopsin trafficking has been studied in great details. Work by Dusanka Deretic, Alecia Gross, and others has revealed conserved mechanisms of rhodopsin trafficking to the base of cilia, including the involvement of small GTPases ARF4, RAB11, and RAB8A and their effectors [22,42,43,44,45]. Given massive OS directed transport in photoreceptors, it was found that the OS indeed is a default destination for membrane proteins in photoreceptors [46]. Nonetheless, distinct mechanisms are employed by some OS membrane proteins to target to cilia. The CNG channel specifically localizes to the ciliary plasma membrane by a mechanism mediated by ankyrin-G [47]. Moreover, peripherin-2 adopts an unconventional secretory pathway involving coatomer subunits COPII to exit from the ER and traffic to the OS [48,49]. These studies further suggest that OS formation and renewal require both conventional and unconventional means of protein delivery. Work from Joe Besharse’s laboratory showed that rhodopsin, guanylate cyclase and chaperone proteins are potential cargo for IFT-mediated delivery into the OS [50].

In addition to IFT, two other ciliary protein complexes have been identified in the cilia. One of them is called BBSome (BBS protein complex) [51,52]. Mutations in the components of the BBSome are associated with multisystem disorders, including retinal degeneration. Work using several model systems has revealed a critical role for the BBSome in regulating retrograde trafficking in cilia. Although a direct involvement of retrograde transport of OS proteins mediated by the dynein motor is lacking, it was found that disruption of cytoplasmic dynein-2 in zebrafish affects OS extension but does not alter the trafficking of opsin or arrestin. Three phototransduction cascade components, arrestin, transducin, and recoverin traffic bidirectionally between the inner and the outer segments in a light-dependent manner in rods [53,54,55]. Such translocation assists in the efficient regulation of the phototransduction cascade by modulating the opsin molecule. It was found that arrestin could diffuse through the TZ into and out of the OS [56,57]. However, molecular mechanisms underlying such transport are not clear. Moreover, this property of bidirectional light-dependent movement of proteins seems highly specific to photoreceptor cilia. A recent report showed that rhodopsin and peripherin-2 are also trafficked preferentially in a light-dependent manner in photoreceptors. Such trafficking is probably linked to variable composition of the OS discs depending upon the time of day [58].

The other protein complex consists of the proteins mutated in syndromic and non-syndromic forms of cystic kidney diseases Nephronophthisis (NPHP) [59]. Different NPHP protein complexes have been localized to distinct ciliary domains [60]. In photoreceptors, there are at least two NPHP protein complexes that are associated with a retinal ciliary disease protein RPGR (retinitis pigmentosa GTPase regulator) [61,62]. Their precise role in regulating photoreceptor cilia function remains to be established.

## 7. Photoreceptor Ciliary Gate

The region between the basal body and the base of the OS is termed the “*ciliary gate*”. This region is also called the transition zone (TZ) because the microtubules of the axoneme transition from a triplet to a doublet conformation [63,64].

Tokuyasu and Yamada noted that the doublet microtubule extensions of the axoneme develop connections with the corresponding ciliary membrane, which appeared denser in electron micrographs [19]. Later studies in *Chlamydomonas* *reinhardtii* and in other species identified these cross-linking structures as Y-linkers in cilia and flagella [65,66]. These structures, in addition to providing a structural support, are also thought to act as barriers for abnormal mixing of the cytosolic and ciliary components.

**Figure 3:**
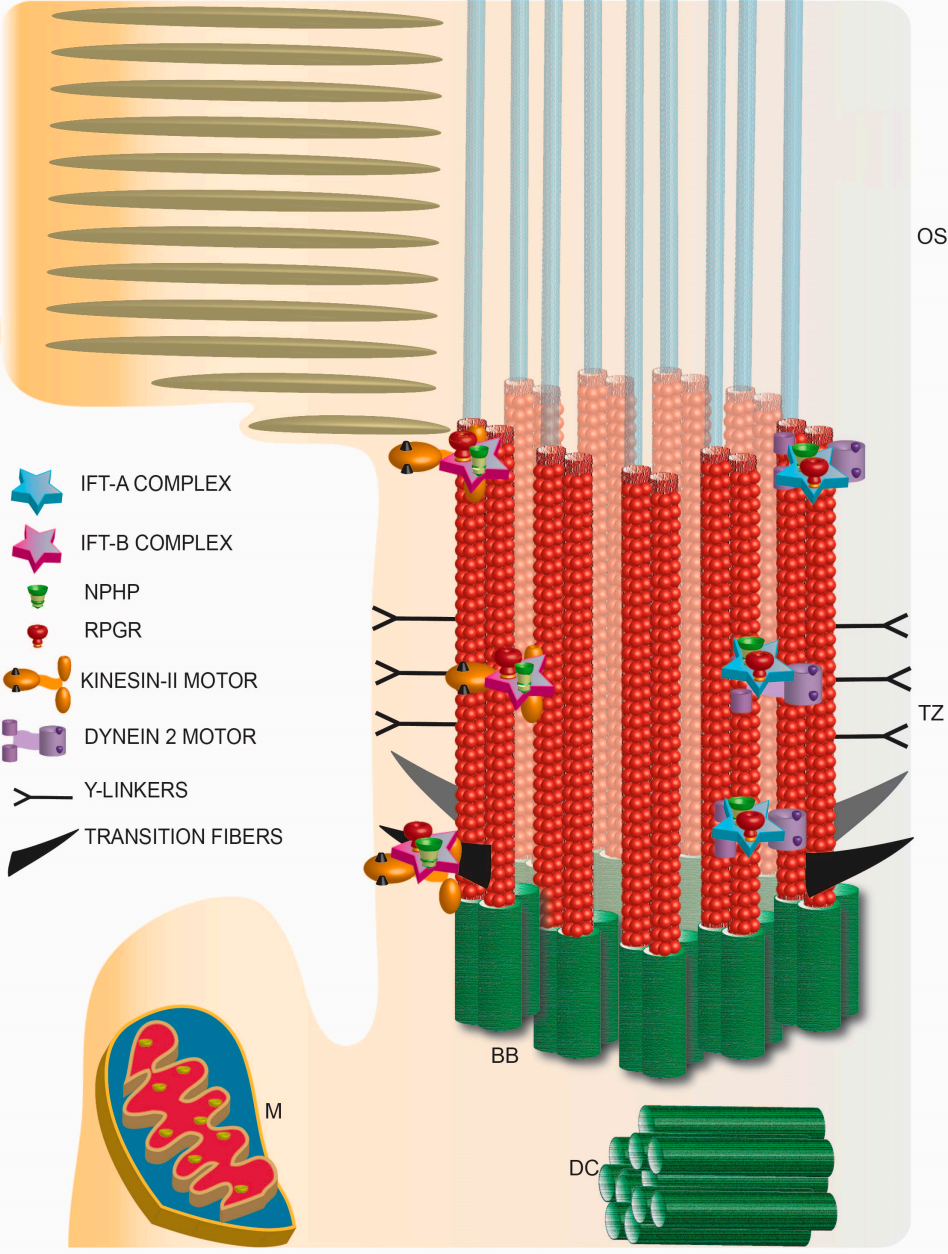
Schematic representation of photoreceptor TZ depicting the identification of the IFT complexes and retinal disease proteins RPGR and NPHP complex proteins. TZ: transition zone; BB: basal body; DC: daughter centriole; M: mitochondria; OS: outer segment.

Additional structures, called Transition Fibers are observed in the region essentially between the basal body and the TZ (Figure 3). During ciliogenesis, the mother centriole develops subdistal and distal appendages. The distal appendages assist in the attachment of the mother centriole to the plasma membrane and become pinwheel shaped transition fibers or alar sheets. Proteins, including OFD1, CCDC123, and CEP164 localize to these structures and are not only involved in the formation of alar sheets but are mutated in human ciliary disorders. Based on their location, transition fibers are thought to be involved in the docking of the vesicles destined to the cilium as they likely provide a physical block to the entry of the vesicles inside the cilium [64]. An analogous structure in frog and rodent photoreceptors was described as the periciliary ridge membrane, which contains a high density of rhodopsin-and IFT-containing vesicles [35,44,67]. These structures are also thought to be composed of Usher syndrome proteins [33]. Based on the function of the constituent proteins, it can be hypothesized that transition fibers are involved in the formation and function of cilia.

## 8. Traversing the Ciliary Gate

Before reaching the OS, the proteins are believed to traverse the narrow TZ (Figure 3). Functional relevance of the TZ in photoreceptors was first noted by Spencer *et al*. (1988) [68]. They found that fusion of frog photoreceptor outer and inner segment results in the redistribution of opsin to the inner segment. These results indicated that rhodopsin is mobile in the OS membrane and that there exists a diffusion barrier to restrict the backward flow of opsin. The TZ contains distinct Y-shaped linkers that form a ciliary necklace around the microtubule and the ciliary membrane. The composition of the TZ was elusive until recently. George Witman and colleagues showed that CEP290/NPHP6, which is mutated in human retinal ciliopathies localizes to the Y-links of *Chlamydomonas* flagella [62,65,69,70,71,72]. Remarkable work by the groups of Michel Leroux, Jeremy Reiter and of Andrew Peterson revealed the presence of multiprotein complexes at the TZ in mammalian and *Caenorhabditis elegans* cilia. These include the Tectonin complex proteins, NPHP, Joubert Syndrome and Meckel-Gruber Syndrome associated proteins [73,74,75].

It is possible that the TZ-associated proteins interact with IFT proteins to regulate the entry and exit of the membrane proteins and lipids inside the cilium. It was found that B9D2/MKS10 interacts with IFT component Fleer. Moreover, a retinal ciliopathy protein Lebercilin (LCA5) associates with the IFT machinery to modulate photoreceptor opsin trafficking [76,77]. Reduced amounts of IFT proteins were also detected in the photoreceptor cilia of mice with targeted ablation of the *Nphp1* gene, which results in opsin mistrafficking and retinal degeneration [78].

*Trafficking of soluble proteins via the TZ:* Although there is ample evidence for a role of the TZ in regulating membrane protein composition of cilia, its involvement as a barrier to the massive translocation in a light-dependent manner, of soluble proteins such as arrestin, transducin and recoverin by diffusion is not well understood. Interestingly, it was found that ectopic expression of photoactivatable GFP results in its free diffusion through the cilium and equilibration between the inner segment and OS of photoreceptors [79]. To test the effect of size of the protein on its access to the OS, the same group used tandem GFP-fusion proteins with increasing number of the GFP moieties. They found that proteins up to ~81 kDa could freely diffuse into the OS, although to a relatively lesser extent than the diffusion of single GFP protein moiety [80]. However, work by Kristen Verhey and colleagues using microinjection of fluorescent dextrans of different molecular weights showed the presence of a size-exclusion barrier that restricts the entry of larger proteins of ~67 kDa into the cilia [81]. Yet another study by Inoue and colleagues, in which they used rapamycin to trap soluble proteins that diffuse into the cilium proposed that the TZ acts as a molecular sieve to restrict the entry of proteins in a size-dependent manner [82]. Whether there is a size exclusion barrier, which acts solely on the basis of size or the three-dimensional conformation of the proteins remains to be established. It should however be noted that soluble proteins that enter the cilium might be associated with other proteins as part of a functional complex. Hence, studies using endogenous proteins in their native conformation are essential to delineate the presence of a barrier at the TZ for soluble proteins. Moreover, cell-type specific differences in the regulation of the barrier cannot be ruled out. This is specifically critical in the case of photoreceptors, which develop membranous discs that can restrict the movement of soluble proteins inside the OS.

It has also been hypothesized that the periciliary region of the cilium is analogous to the nuclear pore complex. Nucleoporins, components of the nuclear pore complex, regulate the entry of soluble proteins into the cilium [81]. A recent study showed that the loss of a TZ-associated retinal disease protein RPGR results in fewer alterations in the membrane protein composition but significant perturbations in the distribution of higher molecular weight soluble proteins in the OS [37]. Given an association of RPGR with distinct NPHP complexes in the retina, it is possible that some TZ protein complexes regulate the entry and retention of soluble proteins into the OS. Future studies are needed to understand the molecular basis of such a gate and its involvement in human diseases.

## 9. Concluding Remarks

The work described above puts primary cilia at the center of a plethora of functions of the retina, particularly photoreceptors. We learnt that photoreceptor cilia possess shared as well as unique features that are responsible for the highly metabolically active processes of these cell types. An aspect that we have not discussed in this article is the involvement of cilia in retinal development. It has been shown that disruptions of several ciliary genes result in perturbed eye development, usually resulting in microphthalmia. It is well known that several IFT and ciliary proteins function during cell division and in the orientation of the cleavage furrow. Hence, elucidation of the role of cilia, ciliary proteins and associated signaling pathways during retinal development will provide novel insights into their broader involvement in development and developmental diseases.

## Abbreviations

OS: outer segment; TZ: Transition Zone; NPHP: Nephronophthisis; RPGR: retinitis pigmentosa GTPase regulator; BB: basal body; GFP: green fluorescent protein; IFT: intraflagellar transport.
